# Divergent subcortical activity for distinct executive functions: stopping and shifting in obsessive compulsive disorder

**DOI:** 10.1017/S0033291715002330

**Published:** 2015-11-06

**Authors:** S. Morein-Zamir, V. Voon, C. M. Dodds, A. Sule, J. van Niekerk, B. J. Sahakian, T. W. Robbins

**Affiliations:** 1 Behavioural and Clinical Neuroscience Institute, University of Cambridge, Cambridge, UK; 2 Department of Psychology, University of Cambridge, Cambridge, UK; 3 Department of Psychology, Anglia Ruskin University, CambridgeUK; 4 Department of Psychiatry, University of Cambridge, Cambridge, UK; 5 Department of Psychology, University of Exeter, UK; 6 South Essex Partnership Trust, UK

**Keywords:** Cognitive flexibility, functional magnetic resonance imaging (fMRI), OCD, response inhibition, shifting, stopping

## Abstract

**Background:**

There is evidence of executive function impairment in obsessive compulsive disorder (OCD) that potentially contributes to symptom development and maintenance. Nevertheless, the precise nature of these executive impairments and their neural basis remains to be defined.

**Method:**

We compared stopping and shifting, two key executive functions previously implicated in OCD, in the same task using functional magnetic resonance imaging, in patients with virtually no co-morbidities and age-, verbal IQ- and gender-matched healthy volunteers. The combined task allowed direct comparison of neural activity in stopping and shifting independent of patient sample characteristics and state variables such as arousal, learning, or current symptom expression.

**Results:**

Both OCD patients and controls exhibited right inferior frontal cortex activation during stopping, and left inferior parietal cortex activation during shifting. However, widespread under-activation across frontal-parietal areas was found in OCD patients compared to controls for shifting but not stopping. Conservative, whole-brain analyses also indicated marked divergent abnormal activation in OCD in the caudate and thalamus for these two cognitive functions, with stopping-related over-activation contrasting with shift-related under-activation.

**Conclusions:**

OCD is associated with selective components of executive function, which engage similar common elements of cortico-striatal regions in different abnormal ways. The results implicate altered neural activation of subcortical origin in executive function abnormalities in OCD that are dependent on the precise cognitive and contextual requirements, informing current theories of symptom expression.

## Introduction

Executive functions enabling suppression or shifting away from no longer relevant actions or thoughts, may be impaired in several neuropsychiatric disorders. In particular, obsessive compulsive disorder (OCD), which is characterized by intrusive distressing thoughts and compulsions (APA, 2013). Whereas stopping or response inhibition involves the deliberate overriding or resisting of dominant responses, shifting refers to the ability to flexibly switch between mental sets or tasks (Miyake *et al.* 2000; Snyder *et al.* 2015). Both may contribute to OCD symptom development and maintenance, with cognitive inflexibility and difficulties in inhibiting unwanted behaviour fostering rigid beliefs and repetitive behaviours that are resistant to change (Chamberlain *et al.* 2005). Indeed, several recent meta-analyses have concluded that despite heterogeneity in the literature, OCD is associated with broad executive function impairments with medium to large effect sizes (Abramovitch *et al.* 2013; Shin *et al.* 2014; Snyder *et al.* 2015). Despite the specific theoretical importance suggested for stopping and shifting in relation to OCD (Chamberlain *et al.* 2005), impairments of broadly similar magnitude were noted for additional executive function sub-domains such as working memory and planning (Abramovitch *et al.* 2013; Snyder *et al.* 2015). Thus, it has been proposed that executive function difficulties in OCD are due to a shared, general overlapping component (Snyder *et al.* 2015).

Evidence from functional imaging can test this hypothesis. Executive function abnormalities are broadly consistent with the fronto-striatal dysfunction reported in OCD (Menzies *et al.* 2008; Milad & Rauch, 2012). However, functional imaging studies of executive functioning in OCD have yielded heterogeneous findings, with limited convergence. Some studies have shown increased prefrontal activation in patients, having attributed this to overactive monitoring (Maltby *et al.* 2005). However, extensive hypoactivation in switching, working memory and spatial planning tasks has also been reported (van den Heuvel *et al.* 2005; Nakao *et al.* 2009). To reconcile such disparities, it was suggested that over-activation may characterize affective ventral corticostriatal systems with hypoactivation in more dorsal, putatively cognitive, circuits (van den Heuvel *et al.* 2005; Nakao *et al.* 2009). However, complex and even contradictory over- and under-activation patterns have been noted in non-affective executive tasks. For example, during response inhibition both OCD-specific increased and decreased activation in the caudate, thalamus and cingulate have been reported (Maltby *et al.* 2005; Roth *et al.* 2007; Page *et al.* 2009; Kang *et al.* 2013).

These seemingly contradictory results have yielded competing interpretations. Increased activation during executive functioning has been interpreted as compensatory (Roth *et al.* 2007; Page *et al.* 2009; de Vries *et al.* 2014) or overactive self-regulation processes (Ursu *et al.* 2003). At the same time, reduced activation was taken to indicate general dysfunction/executive impairment (Remijnse *et al.* 2013) or insufficient recruitment possibly due to interference from chronic OCD symptom-related over-activation (Evans *et al.* 2004). Abnormal activation patterns may also reflect compensatory use of alternate neural substrates in patients (Page *et al.* 2009).

As with behavioural studies, heterogeneous findings can be attributed in part to between-study differences in patient characteristics such as symptom severity, co-morbidities and medication status (Kuelz *et al.* 2004). Additionally, task demands, including difficulty, load, and learning requirements vary considerably between studies, leading to differences in state fluctuations in attention, motivation or even current symptom expression.

To address the competing interpretations regarding functional abnormalities in OCD, we combined different subconstructs from the Research Domain Criteria framework (www.nimh.nih.gov/research-priorities/rdoc/index.shtml) found under cognitive control, in a theoretically driven manner (Miyake *et al.* 2000). Specifically, we contrasted the neural correlates of stopping and shifting within the same task in adult OCD patients compared to matched healthy controls, thus controlling not only for patient-related confounds but importantly also for task-related confounds. This in turn promotes the understanding of the neural networks involved in response inhibition and switching, possibly leading to implications for patients’ symptoms and experience. In healthy adults, this combined task has previously revealed stopping specific activation in the right inferior frontal cortex (IFC) and shifting specific activation in the left inferior parietal cortex (IPC), against a background of extensive co-activation for both in fronto-parietal regions (Dodds *et al.* 2011). In sum, examining two key distinct yet overlapping executive functions (Miyake *et al.* 2000), of likely relevance to OCD symptoms, allowed us to investigate the neurobehavioural specificity in the dysexecutive functioning of patients.

## Method and materials

### Participants

Nineteen OCD patients with median Yale–Brown Obsessive-Compulsive Scale (YBOCS) score of 20 (range 12–30) were matched for age and gender with 19 healthy controls (14 females in each group). OCD patients were recruited from the Cambridgeshire and Peterborough Foundation NHS Trust and from local support groups. Diagnosis according to the DSM-IV criteria followed a detailed interview with a psychiatrist or clinical psychologist supplemented with the MINI (Sheehan *et al.* 1998). The patients did not satisfy DSM-IV criteria for other Axis-I disorders with the exception of two who satisfied criteria for generalized anxiety disorder. Thirteen patients were prescribed serotonin reuptake inhibitors and one a tricyclic antidepressant. Exclusion criteria included substance abuse in the last 3 months and prior diagnosis of schizophrenia, psychotic disorders, bipolar disorder or attention deficit hyperactivity disorder (ADHD). Control participants were recruited via posters in the community and from the Behavioural and Clinical Neuroscience Institute participant panel. Data from three controls were included in a previous report (Dodds *et al.* 2011). For controls, exclusion criteria included no current or past psychiatric disorders and no psychoactive medications. For all participants further exclusion criteria were current or past neurological disorders (including tic disorders), brain damage or magnetic resonance imaging (MRI) contraindications. At testing, the YBOCS (Goodman *et al.* 1989) and Obsessive Compulsive Inventory – Revised (OCI-R; Foa *et al.* 2002) assessed OCD severity, the National Adult Reading Test (NART; Nelson, 1982) assessed verbal IQ and the Montgomery–Asberg Depression Rating Scale (MADRS; Montgomery & Asberg, 1979) assessed depressive symptom severity. The Cambridge Local Research Ethics Committee (08/H0308/65) approved the study, and participants provided informed consent and were reimbursed for participation.

### Procedure

Participants performed a combined shifting go/no-go task (Dodds *et al.* 2011). On each trial they were presented with a superimposed image of a face and a house. The image border colour determined relevant stimulus dimension, for example a red border denoted faces while blue denoted houses as presently relevant. In complex blocks border colour changed every few trials, with that trial constituting a shift trial, where subjects had to shift their attention accordingly between face and house stimuli dimensions. Go/no-go responses were determined by face gender or house storey. For example, participants were told when the border was red they had to attend the faces and respond when the face is female and withhold responding when the face is male (see Supplementary Fig. S1). When the border was blue, they had to attend to houses and respond to two-storey houses but not to one-storey houses. In simple blocks the colour remained constant and subjects attended a single stimulus dimension (faces or houses) throughout. Participants completed a simple and complex block in each of two runs, with block order and go/no-go rules counterbalanced across subjects within each group. On each trial, a red or blue border appeared for 1000 ms, following which the image of an overlapping face and house was presented inside this frame. On go trials, participants had to respond within 725 ms whereupon the display disappeared. On no-go trials, participants had to refrain from responding for the same duration. Following a correct response, a blank screen appeared for 1000 ms, whereupon negative verbal feedback was presented for the same duration following an incorrect response.

Prior to entering the scanner, participants practised both conditions to ensure they understood the task and instructions, which were again presented before each block in the scanner for 10 s, informing participants of the go/no-go and shift rules. In the simple version there were a total of 40 stop and 280 go trials, and in the complex version, there were 40 stop, 40 shift, and 240 go trials, yielding a ratio of stop:go trials and shift:go trials of 1–7. Blocks consisted of approximately 160 trials (158–166), with 4–12 go trials between consecutive stop trials and between consecutive shift trials. The task was presented via E-Prime (Psychological Software Tools Inc., USA) and projected onto a mirror in the scanner, where responses were registered via a customized button box.

### Scanning acquisition

Scanning was carried out at the Wolfson Brain Imaging Centre, Cambridge, on a 3-T Siemens Tim Trio scanner. Functional imaging data were collected in a single session using whole-brain echo planar images (EPI) with the following parameters: repetition time (TR) = 2000 ms; echo time (TE) = 30 ms; flip angle = 78°; 32 slices with slice thickness 3 mm plus 0.75 mm gap; matrix = 64 × 64; field of view (FOV) = 192 × 192 mm yielding 3 × 3 mm in-plane resolution; echo spacing 0.47 ms and bandwidth 2442 Hz/Px. Volumes acquired per run varied from 456 to 485 depending on total trial number. Structural T1-weighted MR scans using a magnetization-prepared rapid acquisition gradient-echo (MPRAGE) sequence were used for registration (176 slices of 1 mm thickness; TR = 2300 ms; TE = 2.98 ms, TI = 900 ms, flip angle = 9°, FOV = 240 × 256 mm).

### Data analysis

For behavioural data, repeated-measures analyses of variance (ANOVAs) contrasted group (OCD *v*. controls) on commission errors and omission errors for each block (simple *v*. complex). Additionally, a 2 × 2 × 3 ANOVA compared group correct go reaction times (RT) for face *v.* house stimuli on simple, complex and switch trials. Functional magnetic resonance imaging (fMRI) data were processed and analysed using Statistical Parametric Mapping 8 (SPM, http://www.fil.ion.ucl.ac.uk/spm/). Images from the first five volumes were discarded to allow for T1 equilibrium effects. Images were slice time-corrected and spatially realigned, and then co-registered to the structural image using the mean functional volume. Subsequent normalization to the Montreal Neurological Institute (MNI) template was followed with re-sampling of EPI volumes to 2 mm isotropic voxels and smoothing with a 6-mm full-width half-maximum Gaussian kernel. Design matrices were implemented using the general linear model (GLM). First-level regressors for complex blocks: correct stop trials, shift trials, and two subsets of correct go trials; for simple blocks: correct stop trials and a subset of correct go trials. Additional regressors of no interest included incorrect stop trials, and parametric modulators for go and shift RT. Go trials comprised separate random selections of trials matched in number to correct stop or shift trials in that block, and were included to allow subsequent conjunction analyses with separate baselines (see Dodds *et al.* 2011 for additional details). Regressors, modelled at target onset, were convolved with a canonical haemodynamic response function. The data were high-pass filtered (1/128-Hz cut-off) and serial correlations were accounted for by a first-degree autoregressive AR (1) model. Mean number of trials was 26, 30 and 34 for complex stop, shift and simple stop contrasts, respectively. Contrasts for each participant for shift *v*. go, stop *v*. go and simple stop *v*. go were used in second-level analyses.

Second-level analyses compared the groups in complex stopping *v*. go, shifting *v*. go, and simple stopping *v*. go in two-sample *t* tests. To investigate common or diverging process-specific abnormalities, we further examined trial/task type and group in second-level whole-brain repeated-measures ANOVAs. Activations associated with general executive functions were investigated with common activations for stopping and shifting using random-effects conjunction analyses against the conjunction null hypothesis. These served as a search area to inspect potential group differences in overall activation in relevant fronto-parietal regions. Divergent abnormalities were examined with the interaction between task and group. All analyses, both between and within groups, were conducted at the whole-brain with family-wise error (FWE) correction set at *p* < 0.05 unless otherwise stated. Secondary uncorrected whole-brain analyses were set to *p* < 0.001 with minimal extent of 5 voxels to provide a more complete overview of the findings and to counteract concerns regarding type II error. Where appropriate, to better characterize results from the above whole-brain analyses, *post-hoc* analyses were conducted on anatomical regions of interest (ROIs; Brett *et al.* 2002), taken from the Automated Anatomical Labeling atlas (Tzourio-Mazoyer *et al.* 2002).

## Results

### Demographics and clinical measures

The groups were matched for age, gender and verbal IQ with OCD patients reporting increased OCD symptom severity levels and slightly elevated depression, although not in the clinical range (see Table 1).

**Table 1.** Demographic and clinical characteristics of OCD and control groups

	Controls		OCD patients			
	Mean	s.d.	Mean	s.d.	*t*	*p*
Age (years)	36.16	11.27	37.79	10.10	0.469	0.469
Verbal IQ	117.44	6.89	114.40	7.92	1.248	0.220
MADRS	3.89	3.05	9.21	6.02	3.431	0.001
YBOCS						
Obsessions			10.47	3.17		
Compulsion			9.47	3.86		
Total			19.95	5.98		
OCI-R	10.89	6.83	26.94	13.65	4.561	0.001
STAI-state	28.74	7.26	40.33	11.56	3.67	0.001
STAI-trait	33.79	9.86	53.06	12.99	5.098	0.001

OCD, Obsessive compulsive disorder; IQ, intelligence quotient; MADRS, Montgomery–Asberg Depression Rating Scale; YBOCS, Yale–Brown Obsessive Compulsive Scale; OCI-R, Obsessive Compulsive Inventory – revised; STAI, State-Trait Anxiety Inventory.

### Behavioural measures

There were no significant behavioural differences between OCD patients and controls in any performance indices. There were more commission errors in the complex (28.26%) compared to the simple (15.05%) tasks, (*F*_1,36_ = 46.60, *p* < 0.001), but no significant group effect (*p* = 0.629) nor did group interact with difficulty (*p* = 0.176). Mean omission errors in simple go trials was 2.76%, in complex go trials 3.47% and in switch trials 5.02% (*F*_2,72_ = 5.75, *p* < 0.01). There was no significant group effect (*p* = 0.218), nor did it interact with trial type (*p* = 0.931). Finally, in an ANOVA with group, trial type and stimulus type as factors, mean RT was 599 ms for controls and 614 ms for patients, which was not significantly different (*F*_1,36_ = 1.11, *p* = 0.298). Responses to faces were faster than to houses (600 *v*. 613 ms, respectively; *F*_1,36_ = 21.947, *p* < 0.001). There was an interaction with trial (*F*_1,36_ = 23.479, *p* < 0.001), with slower RTs to houses compared to faces in complex go trials (*F*_1,36_ = 40.23, *p* < 0.001) and shift trials (*F*_1,36_ = 28.69, *p* < 0.001) but not simple trials (*p* = 0.121). This pattern clearly indicated participants successfully shifted their attention on shift trials to the relevant dimension, and planned comparisons indicated this was the case for both controls (*F*_1,36_ = 16.24, *p* < 0.01) and patients (*F*_1,36_ =  12.57, *p* < 0.01). Additional comparisons of switch costs similarly did not reveal any group differences (*p*'s > 0.31). In sum, no group differences were noted in any analyses. Additionally, no performance indices correlated with OCD or depression severity in the patients. The absence of behavioural group differences means that any changes in fMRI activations below cannot be attributed to performance effects, being more likely to represent underlying neural group differences.

### Neuroimaging

#### Shifting

**Fig. 1.**
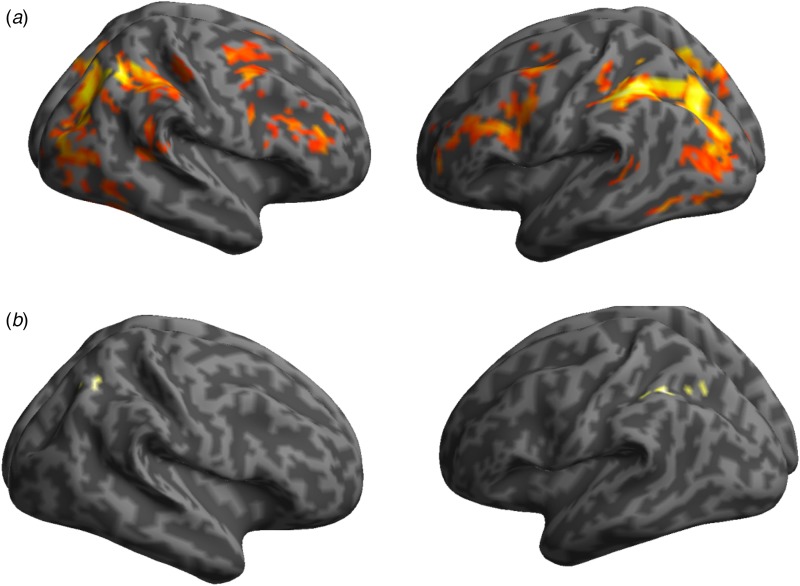
Whole-brain shifting-related activation with a threshold of *p* < 0.05 family-wise error corrected. (*a*) Illustration of fronto-parietal and occipital region activations in a group of healthy control participants. (*b*) Illustration of inferior parietal region activations in a group of obsessive compulsive disorder (OCD) participants.

OCD patients showed lower activation associated with shifting than healthy controls in the left pre-supplementary motor cortex, right precuneus, occipital cortex bilaterally and right thalamus, in a whole-brain analysis corrected at FWE, *p* < 0.05 (see Table 2). There was no evidence for increased activations in the OCD group compared to controls even when lowering the threshold to whole-brain uncorrected *p* < 0.001. When the groups were assessed individually, whole-brain analyses corrected at FWE, *p* < 0.05 showed shifting related activation in controls in fronto-parietal regions, with peaks in the left inferior parietal, IFC bilaterally and striatum in addition to the occipital cortex bilaterally. Patients showed only a few clusters in the left inferior parietal with additional isolated activations in the right parietal lobe (see Fig. 1 and Supplementary material).

**Table 2.** Group differences in brain activation in whole-brain analyses, family-wise error corrected p < 0.05

Contrast	Hemisphere	*Z* score	Peak coordinates MNI (mm)			Cluster size (voxel)	Brain region
			x	y	z		
**Stopping**							
Controls > OCD							
OCD > Controls	L	4.94	−24	−54	22	4	Cuneus
	R	5.20	30	4	32	3	Precentral gyrus
	L	4.77*	−16	6	26	2	Caudate
**Shifting**							
Controls > OCD	L	5.54	−24	−48	−16	15	Fusiform
	R	5.39	48	−74	10	12	Middle temporal
	R	5.33	30	−72	24	17	Middle occipital
	L	5.10	−6	−80	−4	8	Lingual
	L	5.01	−36	−82	−4	6	Middle occipital
	L	4.97	−2	16	50	6	Pre-supplementary motor area
	R	5.00	14	−6	−6	3	Thalamus
	L	4.99	−4	10	60	1	Supplementary motor area
	L	4.87	−30	−78	36	1	Middle occipital
	L	4.84	−26	52	18	1	Middle frontal
	R	4.83	38	−44	−12	1	Fusiform
	R	4.81	26	−58	20	1	Precuneus
	R	4.79	36	−78	18	1	Middle occipital
**Interaction** ^a^							
OCD > Controls	L	5.48	0	−4	8	70	Thalamus
	L	5.37	−12	−6	16		Caudate
	R	5.28	18	−10	−6	7	Thalamus
	R	4.98	12	−4	16	6	Caudate
	L	4.97	−36	−16	30	4	Postcentral gyrus
	R	4.89	40	−56	8	4	Middle temporal gyrus
	R	4.84	28	−58	22	3	Precuneus
	R	4.82	4	−10	12	3	Thalamus
	R	4.81	14	4	32	1	Anterior cingulate
	R	4.72	10	−58	50	1	Precuneus

OCD, Obsessive compulsive disorder; MNI, Montreal Neurological Institute.

Coordinates are in MNI space. All differences are at whole-brain with family-wise error corrected at *p* < 0.05 unless otherwise stated.

a Interaction refers to voxels associated with greater activation in OCD patients compared to controls when stopping but reduced activation in OCD patients compared to controls when shifting.

*Significant at *p* < 0.06 corrected for family-wise error.

#### Stopping

During complex stopping, OCD patients demonstrated greater activation than controls in the left occipital lobe in a whole-brain analysis corrected at FWE, *p* < 0.05 and left caudate, *p* < 0.06 (see Table 2). There was no evidence of hypoactivation in the OCD patients compared to controls, even a threshold of whole-brain uncorrected, *p* < 0.001. When each group was inspected individually, whole-brain analyses corrected at FWE, *p* < 0.05 showed stopping related activations in healthy controls in the IFC bilaterally as well as the parietal and occipital lobes bilaterally. At this threshold, patients demonstrated stopping related activation confined to the right IFC, the parietal cortex bilaterally and the left thalamus in addition to the left occipital lobe (see Supplementary material).

During simple stopping no significant differences were noted between patients and controls. Each group demonstrated significant activation in the right IFC, with patients showing also activation in the left fusiform and controls showing activation in the inferior parietal bilaterally in addition to the right angular gyrus, right fusiform and occipital cortex (see Supplementary material for further analyses).

### Common activation associated with stopping and shifting

**Fig. 2.**
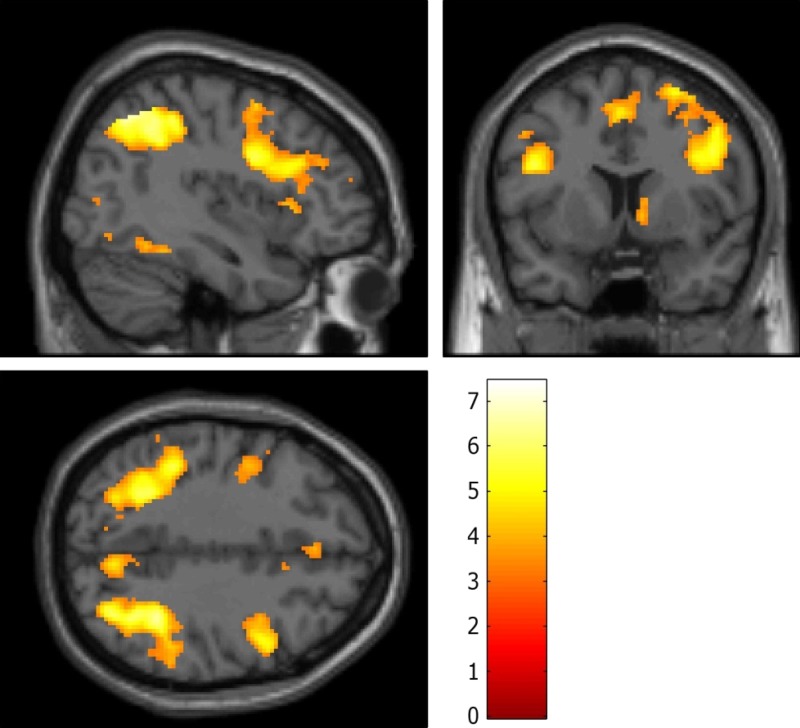
Areas commonly activated during stop and shift trial relative to go trials across all participants overlaid on the MNI brain. Images are displayed at x = 40, y = 8 and z = 38 in the sagittal, coronal and axial planes, respectively, with a voxel-wise threshold of *p* < 0.001 uncorrected. Colour bar represents *t* scores.

The conjunction whole-brain analyses corrected at FWE, *p* < 0.05 across all individuals revealed fronto-parietal activations during stop and shift trials compared to go trials. These areas included clusters in the inferior parietal cortex bilaterally, in addition to IFC bilaterally, left supplementary motor area, left fusiform gyrus, left middle occipital gyrus, right precuneus and right middle and superior and frontal gyri (Fig. 2). Overall mean activation across this search area was significantly reduced in the OCD group compared to controls for shifting (*t*_35_ = 3.17, *p* < 0.001) but not stopping (*t*_35_ = 0.57, *p* > 0.701). These results survived when the conjunction-based search area was defined by a more liberal threshold (*p* < 0.001 uncorrected) or an independent search area (Morein-Zamir *et al.* 2014).

### Opposing abnormal activation associated with stopping and shifting

**Fig. 3.**
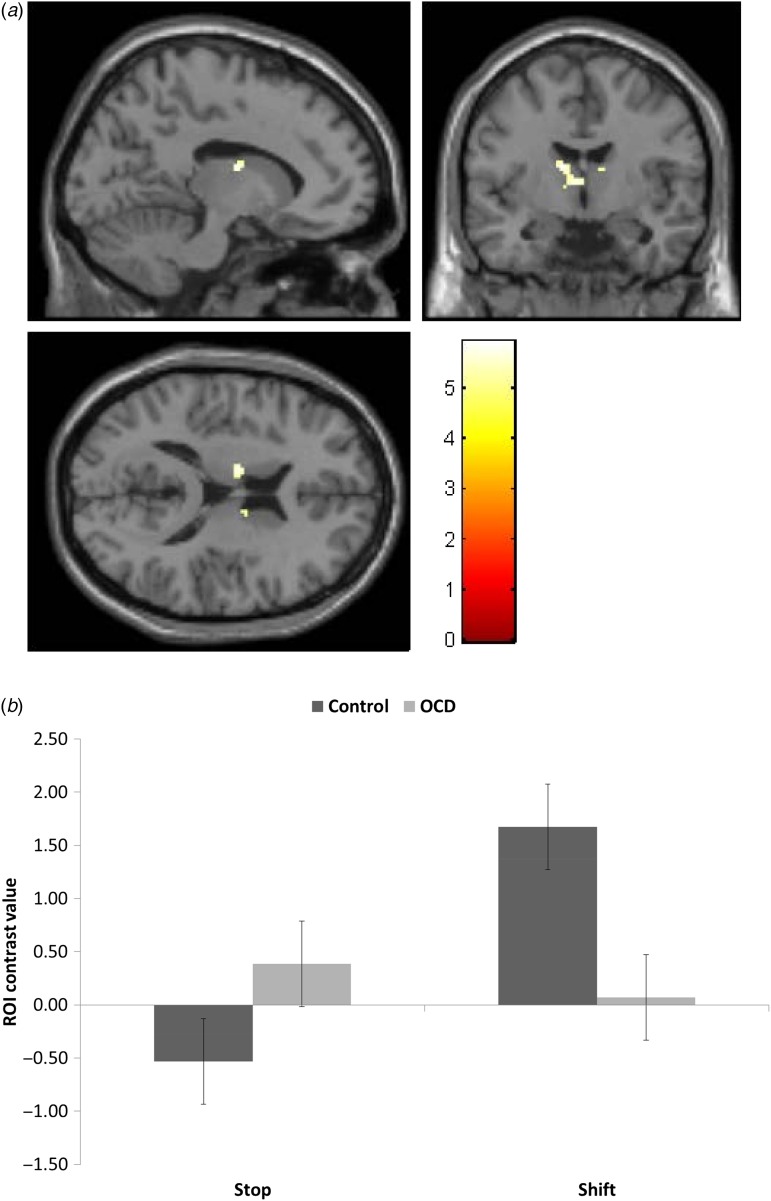
Voxels associated with greater activation in obsessive compulsive disorder (OCD) patients compared to controls when stopping but reduced activation in OCD patients compared to controls when shifting. (*a*) Areas showing this pattern of activation are displayed overlaid on the MNI brain. Images are displayed at x = −12, y = −6 and z = 16 in the sagittal, coronal and axial planes, respectively, with a voxel-wise threshold of *p* < 0.05 family-wise error corrected. Colour bars represent *t* scores. (*b*) Region-of-interest *post-hoc* analysis of activity for stop and shift trials in control and OCD patients groups in the caudate bilaterally. Error bars represent s.e.m..

We also investigated whether there were brain regions associated with opposing activations during stopping and shifting in the OCD patients compared to controls, using a mixed-measures ANOVA with stopping and shifting as repeated measures and group as a between-subjects measure. As noted in Table 2, whole-brain analyses corrected at FWE, *p* < 0.05 indicated significant activations in the thalamus and caudate bilaterally, in addition to the right precuneus, with patients showing increased activation for stopping but decreased for shifting compared to controls (Fig. 3*a*). To better characterize the interaction in the caudate observed in the whole-brain analysis, individual contrast values were derived from caudate anatomical ROIs and entered into a mixed-measures ANOVA with group, trial type and side as independent variables. Though no main effect of group, there was a group×task interaction (*F*_1,36_ = 23.73, *p* *<* 0.001). Comparisons indicated greater bilateral caudate activation in controls compared to patients for shifting (*F*_1,36_ = 13.61, *p* = 0.001) and greater bilateral caudate activation in patients compared to controls for stopping (*F*_1,36_ = 5.23, *p* = 0.028) (Fig. 3*b*). Correlation analysis revealed greater symptom severity was associated with reduced caudate activation during shifting, (*r* = −0.41, and −0.43, *p* < 0.08, for left and right caudate, respectively).

## Discussion

In a combined stop-shift task, OCD patients with virtually no co-morbidities engaged broadly the same regions as healthy volunteers, with right IFC activations during stopping and left IPC activations during shifting. Importantly, however, extensive *under-activation* specifically during shifting was found for patients compared to controls across fronto-parietal regions associated with executive functioning. During stopping patients exhibited focal *over-activation* in the caudate and thalamus and the medial occipital lobe. The caudate and thalamus, regions previously implicated in OCD, showed contrasting patterns of abnormality in the patients using conservative whole-brain analyses. The under-activation during shifting, showing some association with symptom severity in the caudate, and the opposing over-activation of this same region during stopping, support fronto-striatal abnormalities in OCD, while also clearly implicating fronto-parietal regions in aspects of executive dysfunction. In contrast to the hypothesis that there would be overlapping abnormalities between stopping and shifting, indicative of general executive impairment in OCD, the findings point to multiple distinct neural correlates of executive abnormalities.

The opposing aberrant activations demonstrate how observed functional abnormalities in OCD depend on the precise cognitive requirements with no tendency for general task-related ‘hypoactivation’ or ‘hyperactivation’ of key structures. This may have implications more generally for the interpretation of imaging findings in OCD, as it underlines how not only cognitive demands but also symptom provocation and task challenges may yield opposite activations in regions such as the caudate or orbitofrontal cortex (Milad & Rauch, 2012). Present findings also help clarify the inconsistent imaging results reported, purporting that seemingly opposing findings for a given neural region in different settings could be a key characteristic of fronto-striatal OCD dysfunction. Participants were required to inhibit responding and shift attention in the same task, ruling out a host of situational variables (e.g. on-task symptom expression, fatigue, and practice) that could underlie between-task or between-study differences. Similarly, shifting and stopping did not differ in important task demands, with neither requiring trial-and-error learning, and both occurring equally infrequently, rendering salience or attentional capture unlikely to account for the results. For both stopping and shifting, instructions left no ambiguity regarding which was the appropriate response on each trial. Such factors have likely contributed to inconsistencies in findings (Morein-Zamir *et al.* 2013*b*). The results caution against simple models of executive impairment in OCD and against attributing global over- or under-activation to particular circuits. Inhibitory dysfunction is thus unlikely simply to result from hypoactive brain regions associated with cognitive control and/or overactive brain areas associated with error monitoring (Page *et al.* 2009; de Wit *et al.* 2012). Similarly, caudate and thalamus overactivation during stopping indicates that their hypoactivation during shifting is not due to these regions being generally less capable of recruitment. Rather, present findings indicate that neural abnormalities associated with executive function in OCD appear, as a rule, task-dependent.

The hypoactivation associated with rule-determined shifting demonstrates insufficient widespread recruitment for this cognitive function, lending credence to former findings (Gu *et al.* 2008; Page *et al.* 2009). In contrast to a previous study reporting no shift-related activation in OCD patients (Gu *et al.* 2008), we noted left IPC activation, albeit at a reduced level (see Supplementary material). This region, also found in the controls, is implicated in switching or shifting (Wager *et al.* 2004), suggesting that patients utilize the relevant neural substrates, although insufficiently so. Reduced brain activation despite adequate task performance is commonly observed in OCD (Maltby *et al.* 2005; Nakao *et al.* 2005; Page *et al.* 2009), supporting the suggestion that it may be a sensitive index of neurocognitive dysfunction even in the absence of behavioural differences. This is in line with the notion that performance in cognitive flexibility tasks where correct responding is determined by explicit rules may be insensitive to the commonly reported inflexibility and perfectionism (Moritz *et al.* 2004; Meiran *et al.* 2011). At the same time, the tentative association between symptom severity and reduced caudate activation links inefficient recruitment during shifting to a key brain region implicated in the disorder. The widespread hypoactivation may also relate to difficulties in late-stage disengagement reported in OCD (Morein-Zamir *et al.* 2010, 2013*a*) providing a more specific delineation of cognitive inflexibility.

In contrast to the widespread shift-related under-activation, stop-related over-activation was largely specific to the caudate and thalamus in whole-brain analyses, both previously implicated in response inhibition. This conforms with abnormal response control in OCD involving fronto-striatal loops (Menzies *et al.* 2008). The caudate and thalamus are widely implicated in OCD pathophysiology including anatomical abnormalities (Rotge *et al.* 2009; Shaw *et al.* 2015) and aberrant functionality during rest, provocation and task performance (Whiteside *et al.* 2004; Rotge *et al.* 2008). The patients also demonstrated increased cuneus activation during stopping. Although unexpected, cuneus hyperactivation in OCD during working memory has been reported (Nakao *et al.* 2009), as has hyperactivation during stopping in the occipital cortex (Roth *et al.* 2007; Page *et al.* 2009). This could reflect heightened processing due to exaggerated emotional responsiveness and arousal or be indicative of compensatory mechanisms allowing adequate performance (Page *et al.* 2009). The results stress the importance of whole-brain analyses and the role of posterior areas in mediating abnormal cognitive function in OCD (Menzies *et al.* 2008).

Prefrontal activation, particularly right IFC, was noted in both groups during stopping with no hypoactivation in OCD, consistent with some studies (Maltby *et al.* 2005; Page *et al.* 2009) but not others adopting ROI or liberal approaches (Roth *et al.* 2007; de Wit *et al.* 2012). Variable prefrontal cortex findings in OCD may result from its prolonged developmental trajectory along with formation of compensatory cognitive strategies and patients’ generally high level of cooperation and motivation. This interpretation is consistent with the adequate performance levels noted. This was advantageous as the brain activation results were not confounded by performance differences (Frith *et al.* 1995; Weinberger & Berman, 1996). Response inhibition deficits are observed in OCD when inhibitory demands are high, but not when they are lower as in go/no-go tasks (Watkins *et al.* 2005; Menzies *et al.* 2007; Bohne *et al.* 2008; Morein-Zamir *et al.* 2010). The present task was not designed to be challenging, employing considerable practice and clear instructions, though it is anticipated that with additional demands, behavioural impairments would have become apparent (Morein-Zamir *et al.* 2013*b*). Further, participants responded within a limited time-window, which may have facilitated performance particularly in OCD patients. In any case, the findings point to a role for subcortical functional integrity during response inhibition in OCD, which may manifest during challenging situations encountered in everyday life. In sum, even a conservative interpretation of present results implicates aberrant striatal and thalamic functioning in OCD during executive functioning.

The results also delineate the advantage of using similar functional paradigms across psychiatric disorders. Fronto-striatal abnormalities and stopping and shifting impairments, have also been implicated in drug dependence, schizophrenia and ADHD (Willcutt *et al.* 2005; Robbins, 2007). Executive function difficulties appear similar between disorders, although direct between-group comparisons are often hindered by sample confounds including age, medication and co-morbidity status. How then can seemingly similar cognitive difficulties contribute to dysfunction in disorders with such disparate symptoms? For example, inhibitory difficulties in ADHD and stimulant users have been linked to an impulsive style, in contrast to OCD (Morein-Zamir & Robbins, 2014). Present results illustrate how neurobiological differences can inform this issue: as opposed to the caudate hyperactivation in OCD, stop-related caudate hypoactivation was found in ADHD children and occasional stimulant users (Rubia *et al.* 2005, 2011; Harle *et al.* 2014). Moreover, a recent study of adult ADHD using the same stop-shift task in our group found strikingly different results to those reported here for OCD. Whereas performance was impaired and abnormalities noted in the right IFC, no shift-related under-activation or stop-related over-activation was observed (Morein-Zamir *et al.* 2014). Similar right IFC under-activation in chronic stimulant users was also reported in a stop-signal task (Morein-Zamir *et al.* 2013*c*). We speculate that stopping abnormalities in OCD are more closely linked to response control aberrations, being less attributable to attentional or executive function difficulties. As such, though subtle behaviourally (Abramovitch *et al.* 2013), stopping abnormalities could contribute to and result from executing deliberate repetitive actions over many years. Taken together, the results demonstrate that, although the neural circuitry and cognitive processes mediating various neuropsychiatric disorders overlap, the disparate clinical features are accompanied by highly distinct functional abnormalities, particularly in the striatum (Hart *et al.* 2013; Shaw *et al.* 2015).

This study employed a well-characterized sample of mixed gender and medication status with almost no co-morbid Axis-I disorders, including depression. Whilst secondary analyses suggested medication was unlikely to play a role (see Supplementary material) as does the evidence from first degree siblings (Chamberlain *et al.* 2008), present sample size was not sufficiently large to address this definitively and future studies should verify the role of medication directly. Similarly, the sample did not allow for analyses regarding symptom dimensions, though patients reported increased symptom severity for all OCI-R subscales except hoarding. The study design did not include null events or rest conditions and so group differences in go trials could not be verified. OCD patients, however, appear to have abnormal resting state activation (Whiteside *et al.* 2004) and therefore inclusion of such conditions could have limited utility. At the same time, the study has several key strengths including examining multiple executive functions within the same task, allowing control of state variables and task demand confounds and employment of conservative whole-brain analyses. In summary, executive dysfunction in OCD appears to be mediated by separable cognitive functions, each associated with distinct patterns of abnormality not only across but also within the same cortico-striatal substrates. The latter finding suggests a new perspective for interpreting the neural substrates of OCD.

## Supplementary material

For supplementary material accompanying this paper visit http://dx.doi.org/10.1017/S0033291715002330. click here to view supplementary material
