# Supplementary material for: Divergent subcortical activity for distinct executive functions: stopping and shifting in obsessive compulsive disorder
Source: Psychol Med. 2015 Nov 6;46(4):829–40. doi: 10.1017/S0033291715002330 (PMC4754830; doi:10.1017/S0033291715002330)
Supplement: Supplementary file 1 [file S0033291715002330sup001.docx]

**SUPPLEMENTAL MATERIAL**

*Effects of medication and depression severity levels on group results*

We examined whether the group effects observed in the caudate were influenced by higher depressive symptom severity in the patients (see Table 1). The ANOVA on caudate activation was repeated with MADRS scores added as a covariate. The interaction remained highly significant (*F*(1,35)=25.39, *p*=0.001). Again there was greater activation in controls compared with patients for shifting (*F*(1,35)=13.031, *p*=0.001) and greater activation in patients compared with controls for stopping (*F*(1,35)=6.07, *p*=0.019). Additionally, we examined whether depressive severity underlined the reduced activation in patients in the executive function fronto-parietal areas associated with shifting. Activation across this search area remained reduced in the OCD group compared with controls for shifting (*F*(1,34)=20.47, *p*<0.001). Although both increased anxiety and lower mood can be considered as core symptoms of the disorder, we further examined correlations between ROI effect sizes and STAI and MADRS scores and noted no significant correlations within the patient group.

We also compared unmedicated and medicated patients directly to assess differences in caudate activation as well as activation across fronto-parietal executive areas. No significant group effects emerged (p>0.55 for all comparisons). As the comparison was between two small groups, Cohen’s d effect size measure was computed. In the caudate stopping related activation was associated with a Cohen’s d of 0.01, and shifting related activation was associated with a Cohen’s d of 0.23 between the medicated and unmedicated subgroups. Shifting related activation was associated with an effect size of 0.08 in the fronto-parietal search area. Taken together it appears that the group differences reported above are unlikely to have been driven by increased depression severity or medication in the OCD patient group.

*Investigating shifting in the inferior parietal cortex*

Prior studies using the stop-shift task reported greater activation for shifting than stopping in the left inferior parietal cortex (IPC) (Dodds *et al*., 2011). To ascertain whether this was presently the case, mean effect sizes were computed for this anatomical ROI in the same method as described in the main text (Tzourio-Mazoyer *et al*., 2002). Significantly greater activation for shift compared to stopping was noted in the control group (*t*(18)=4.45, *p*<0.001) and separately in the OCD patient group (t(18)=2.40, *p*=0.011). Nevertheless, controls demonstrated greater shifting versus stopping compared to the patients in this ROI (*t*(36)=1.92, *p*=0.031). Thus, while both groups demonstrate greater activation for shifting compared to stopping activity in the left IPC, there was also general hypoactivation during shifting in the patients.

*Investigating stopping in complex and simple blocks*

Activations common to both complex and simple stops compared to go trials were investigated using random effects conjunction analyses against the conjunction null hypothesis. These served as a search area to inspect potential group differences in overall activation related to stopping. The conjunction whole brain analyses corrected at FWE p<0.05 across all individuals revealed clusters with peaks in the right IFC (peak coordinate=[32, 24,6], cluster extent (K_E_)=120, Z=6.50), the left IFC ([-32, 22,0], K_E_=84, Z=5.95) as well as the right inferior cortex ([34, -54,48], K_E_=482, Z=6.10) and right precentral gyrus ([46, 8,30], K_E_=24, Z=4.97). Additionally there was small activations in the left pre-supplementary motor area ([-6,12,48], K_E_=4, Z=5.07), right anterior cingulate ([8,14,42], K_E_=1, Z=4.67), right superior temporal lobe ([56,-40,18], K_E_=1, Z=4.67), left occipital lobe ([-40,-88,14], K_E_=12, Z=5.34) and fusiform gyrus ([-34,-46,-18], K_E_=6, Z=5.18). Using the results of the conjunction as a search area did not reveal any significant differences between the OCD and controls groups for complex stopping (*t*(36)=0.44, p=0.667) or simple stopping (*t*(36)=0.44, p=0.668). These results remained when the conjunction-based search area was defined by a more liberal threshold (p<0.001 uncorrected) and with a search area defined in a previous study (Morein-Zamir *et al.*, 2014). Taken together, these results reaffirm the findings put forth in the main text regarding limited evidence for stop-related group differences in fronto-parietal regions. Note that subcortical structures such as the caudate and thalamus were not found in the conjunction and thus these results do not speak to any OCD-related abnormalities in these regions.

**Table S1**. Group activations relating to complex and simple stopping in whole brain analyses, family-wise error corrected p<0.05.

| Task | Group | Hemisphere | Z-score | Peak coordinates MNI (mm) | | | Cluster size (voxel) | Brain Region |
| --- | --- | --- | --- | --- | --- | --- | --- | --- |
|  |  |  |  | x | y | z |  |  |
| Complex stop>go | Control |  |  |  |  |  |  |  |
|  |  | R | 5.64 | 32 | 24 | 4 | 29 | Anterior insula |
|  |  | L | 5.50 | -48 | -40 | 44 | 13 | Inferior parietal |
|  |  | R | 5.50 | 36 | -60 | 48 | 36 | Angular gyrus |
|  |  | L | 5.47 | -30 | 22 | 2 | 55 | Anterior insula |
|  |  | L | 5.09 | -36 | -54 | 42 | 1 | Inferior parietal |
|  |  | L | 5.02 | -40 | -88 | 14 | 1 | Middle occipital gyrus |
|  |  | L | 4.84 | -26 | -66 | 32 | 1 | Middle occipital gyrus |
|  |  | R | 4.81 | 32 | -72 | 34 | 1 | Middle occipital gyrus |
|  | OCD | L | 5.76 | -38 | -90 | 16 | 7 | Middle occipital gyrus |
|  |  | R | 5.60 | 30 | 4 | 32 | 5 | Inferior operculum |
|  |  | R | 5.02 | 32 | -52 | 48 | 4 | Inferior parietal |
|  |  | L | 5.00 | -36 | -56 | 42 | 5 | Inferior parietal |
|  |  | L | 4.96 | 0 | -12 | -2 | 3 | Thalamus |
|  |  | R | 4.80 | 34 | -58 | 48 | 1 | Angular gyrus |
| Simple stop>go | Control | R | 5.61 | 24 | -46 | -10 | 14 | Lingual gyrus |
|  |  | R | 5.45 | 30 | -62 | 40 | 74 | Angular gyrus |
|  |  | R | 5.39 | 38 | -56 | 48 |  | Angular gyrus |
|  |  | L | 5.05 | -42 | -48 | 46 | 15 | Inferior parietal |
|  |  | R | 4.98 | 30 | -62 | 48 | 4 | Angular gyrus |
|  |  | R | 4.96 | 58 | -40 | 26 | 3 | Supramarginal gyrus |
|  |  | R | 4.90 | 38 | 18 | 4 | 5 | Anterior insula |
|  |  | R | 4.84 | 34 | -38 | -18 | 1 | Fusiform gyrus |
|  |  | R | 4.81 | 50 | -38 | 48 | 2 | Inferior parietal |
|  |  | R | 4.80 | 46 | 8 | 34 | 1 | Precentral gyrus |
|  | OCD | L | 5.17 | -34 | -44 | -18 | 2 | Fusiform gyrus |
|  |  | R | 4.98 | 38 | 20 | 8 | 7 | Anterior insula |

**Figure S1:** Illustration of a series of trials in the complex condition. Stimuli consist of superimposed images of faces and houses. In this instance, a red border indicates faces are the relevant dimension with responding to female and withholding responding to male faces, whereas a blue border indicates houses are the relevant dimension with responding to two-story houses and withholding responding to one-storey houses. Trial type is delineated under each stimulus. The inset describes individual trial structure, with an initial blank frame (1000 msec) followed by the stimulus (725 msec) and either a blank frame following correct responding or feedback following incorrect responding (1000 msec).


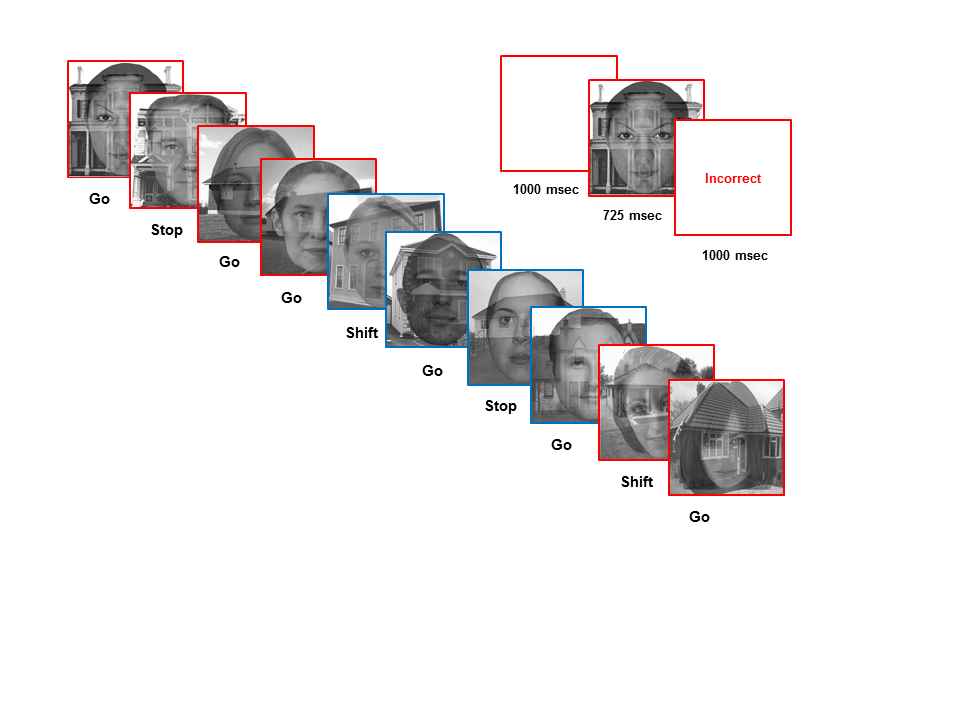


**Supplementary References**

**Dodds, C. M., Morein-Zamir, S. & Robbins, T. W.** (2011). Dissociating inhibition, attention, and response control in the frontoparietal network using functional magnetic resonance imaging. *Cerebral Cortex* **21**, 1155-65.

**Morein-Zamir, S., Dodds, C., van Hartevelt, T. J., Schwarzkopt, W., Sahakian, B. J., Muller, U. & Robbins, T. W.** (2014). Hypoactivation in Right Inferior Frontal Cortex is Specifically Associated With Motor Response Inhibition in Adult ADHD. *Human Brain Mapping* **35**, 5141-52.

**Tzourio-Mazoyer, N., Landeau, B., Papathanassiou, D., Crivello, F., Etard, O., Delcroix, N., Mazoyer, B. & Joliot, M.** (2002). Automated anatomical labeling of activations in SPM using a macroscopic anatomical parcellation of the MNI MRI single-subject brain. *Neuroimage* **15**, 273-89.
